# The Implications of Radiotherapy-Induced Cellular Senescence for Cancer Treatment and Tumor Microenvironment Modulation

**DOI:** 10.7150/ijbs.130683

**Published:** 2026-03-17

**Authors:** Chao Jiang, Zhouting Tuo, Dilinaer Wusiman, Jie Wang, Haohao Yao, William C. Cho, Zhaojie Lyu, Qi Zhang, Dechao Feng

**Affiliations:** 1 Urology & Nephrology Center, Department of Urology, Zhejiang Provincial People's Hospital (Affiliated People's Hospital), Hangzhou Medical College, Hangzhou 310014, Zhejiang, China.; 2 Department of Urology, The Second Affiliated Hospital of Anhui Medical University, Hefei 230601, Anhui, China.; 3 Department of Urology, Tianjin Institute of Urology, The Second Hospital of Tianjin Medical University, Tianjin 300211, China.; 4 Department of Comparative Pathobiology, College of Veterinary Medicine, Purdue University, West Lafayette, IN, 47907, USA.; 5 Purdue Institute for Cancer Research, Purdue University, West Lafayette, IN, 47907, USA.; 6 Department of Clinical Oncology, Queen Elizabeth Hospital, Hong Kong SAR, China.; 7 Department of Urology, Institute of Precision Medicine, Shenzhen Key Laboratory of Male Reproduction and Genetics, Peking University Shenzhen Hospital, Shenzhen 518036, Guangdong, China.; 8 Division of Surgery & Interventional Science, University College London, London W1W 7TS, UK.

**Keywords:** Radiotherapy, Cellular senescence, Senescence-associated secretory phenotype, Tumor microenvironment, Reactive oxygen species, Senolytics

## Abstract

Radiotherapy is a cornerstone of cancer management, yet ionizing radiation can induce cellular senescence in tumor cells and in normal or stromal compartments. Senescent cells undergo durable cell-cycle arrest but remain metabolically active and develop the senescence-associated secretory phenotype (SASP), a bioactive secretome that remodels the tumor microenvironment (TME). This review summarizes principal pathways that couple radiation injury to senescence, including persistent DNA damage signaling, oxidative stress driven by reactive oxygen species, telomere dysfunction, and epigenetic reprogramming, and discusses their downstream consequences. We highlight the time- and context-dependent nature of senescence: early after treatment, senescence can constrain proliferation and enhance immune surveillance; when senescent cells persist, chronic SASP signaling can promote extracellular matrix remodeling, angiogenesis, immune dysfunction, cellular plasticity, and ultimately recurrence and therapy resistance, while also contributing to late normal-tissue toxicity and fibrosis. Finally, we evaluate emerging therapeutic strategies to modulate this biology, including senolytics that eliminate senescent cells, senomorphics that attenuate harmful SASP outputs, immunotherapeutic approaches that augment senescence surveillance, and optimization of radiotherapy delivery to limit normal-tissue senescence. Defining robust biomarkers, treatment windows, and safety profiles will be essential for translating senescence-targeted combinations into durable tumor control with reduced long-term toxicity.

## Introduction

Radiotherapy is a cornerstone of cancer therapy, incorporated into more than half of all treatment plans for malignant tumors worldwide 1. Its therapeutic effect primarily relies on the biological consequences of ionizing radiation, which induces severe DNA damage, particularly double-strand breaks, ultimately resulting in tumor cell death or proliferative arrest 2, 3. In recent years, advances in intensity-modulated radiotherapy (IMRT), proton therapy, and ultra-high-dose-rate FLASH radiotherapy (FLASH-RT) have significantly improved tumor targeting accuracy and dose distribution conformity 4-6. Beyond geometric precision, these modalities also reshape how dose is delivered in space and time, including dose heterogeneity, the extent of low-dose exposure to surrounding tissues, integral dose, linear energy transfer (LET)/relative biological effectiveness (RBE) patterns, and dose rate. These delivery features are increasingly recognized as potential determinants of the burden and anatomical distribution of therapy-induced senescent cells and their SASP in both tumors and adjacent normal tissues. However, unintended irradiation of adjacent normal tissues remains a major limitation, often resulting in severe acute and long-term toxicities. These complications not only affect patients' quality of life but also constrain the maximum tolerable dose of radiation. Clinically, this biology corresponds to three persistent challenges in radiotherapy: (i) incomplete local control leading to recurrence, (ii) dissemination and distant metastasis enabled by microenvironmental remodeling, and (iii) late normal-tissue toxicities—such as fibrosis and organ dysfunction—that constrain dose escalation. Consequently, a mechanistic understanding of therapy-induced senescence and its SASP, along with tools for its measurement and modulation, is directly pertinent to enhancing both tumor control and long-term patient outcomes.

In this context, cellular senescence has garnered increasing research attention. It refers to a state of permanent cell cycle arrest in which cells remain metabolically active and exhibit profound phenotypic alterations 7. Senescence typically occurs in response to cellular stress, particularly extensive DNA damage, and serves as an important tumor-suppressive mechanism by preventing the proliferation of damaged or potentially malignant cells 8. A hallmark feature of senescent cells is the SASP, characterized by the secretion of various bioactive molecules such as pro-inflammatory cytokines, chemoattractants, matrix metalloproteinases, and growth factors 9. These mediators exert complex and context-dependent effects on neighboring cells, thereby remodeling the local tissue microenvironment.

Radiation-induced cellular senescence complicates treatment outcomes by affecting both tumor cells and surrounding normal tissues 10. Specifically, radiotherapy-induced senescence can significantly alter the tumor microenvironment, thereby influencing tumor progression, immune response, treatment resistance, and potential metastatic processes 11, 12. Although the initial induction of senescence may inhibit the proliferation of damaged tumor cells, SASP components can promote tumor recurrence or metastasis through mechanisms such as chronic inflammation and angiogenesis 13-15. This dual role poses challenges for the development of therapeutic strategies.

Given this complexity, systematic investigation into the role of radiotherapy-induced senescence in cancer treatment is of profound importance. A deeper understanding of the molecular pathways underlying senescence and their impact on tumor biology and microenvironmental remodeling may provide critical theoretical foundations for optimizing radiation treatment plans 16. Furthermore, elucidating these mechanisms will facilitate the development of novel combination therapies aimed at mitigating senescence-associated adverse effects 17, thereby improving overall treatment efficacy and patient prognosis. Therefore, exploring the biological significance of radiation-induced cellular senescence represents not only a major focus in current oncology research but also a translationally valuable avenue for refining comprehensive cancer treatment paradigms and enhancing clinical strategies.

## Underlying mechanisms of radiotherapy-induced cellular senescence

This section summarizes the core damage-sensing and stress-response pathways that couple ionizing radiation to stable senescent arrest and SASP activation, thereby establishing a mechanistic foundation for the phenotypes discussed in Sections 3-4.

### DNA damage response (DDR): p53 pathway activation and the role of the p16(INK4a)/Rb pathway

Radiotherapy exerts its anti-tumor effects primarily by inducing substantial DNA damage, particularly double-strand breaks (DSBs) 18-20. Upon the occurrence of DSBs, cells initiate a complex signaling network known as the DNA damage response (DDR), which involves the rapid recruitment and activation of protein kinases such as ATM (ataxia telangiectasia mutated) and ATR (ATM and Rad3-related) to DNA lesion sites 21. Central to the DDR signaling cascade is the tumor suppressor protein p53, often referred to as the “guardian of the genome”. Upon activation, p53 initiates transcriptional programs that lead to DNA repair, cell cycle arrest, apoptosis, or senescence 22. Particularly under conditions of persistent or irreparable DNA damage, sustained p53 activation induces the expression of cell cycle inhibitors, notably p21, which inhibits proliferation by arresting cells at the G1/S transition and promotes the senescence process 23, 24. Additionally, radiation-induced DNA damage activates the p16(INK4a)/retinoblastoma (Rb) pathway—another key regulator of cell cycle progression 25. Activation of p16(INK4a) results in hypophosphorylation of Rb, preventing E2F transcription factors from initiating the expression of genes involved in DNA synthesis, thereby enforcing permanent growth arrest 25.

### Generation of reactive oxygen species (ROS)

Exposure to ionizing radiation leads to substantial generation of reactive oxygen species (ROS) 26. These highly reactive molecules can induce oxidative stress, damaging critical cellular macromolecules including DNA, proteins, and lipids 27. ROS-induced oxidative DNA damage commonly manifests as single-strand breaks, base oxidation, and the more detrimental double-strand breaks 28. Sustained exposure to high ROS levels exacerbates DNA damage, which further amplifies the DDR pathway and drives a stable senescent program 29. Nonetheless, senescence escape has been documented in some tumor contexts, a process that may contribute to relapse. Beyond direct DNA damage, ROS also modulate signaling pathways related to cell growth, apoptosis, and inflammation, thereby contributing to the establishment and maintenance of the senescent phenotype 29, 30. Elevated ROS levels can regulate key transcription factors and signaling molecules such as nuclear factor κB (NF-κB), mitogen-activated protein kinase (MAPK), and phosphoinositide 3-kinase/protein kinase B (PI3K/Akt), subsequently influencing the expression of SASP factors 31. Persistent ROS signaling not only sustains the senescent state but also actively shapes the tumor microenvironment, modulates inflammatory responses and immune activity, and may even promote pro-tumorigenic processes 32, 33. Given the central role of ROS 34, strategies aimed at modulating ROS levels may represent novel therapeutic avenues for modulating radiation-induced senescence and associated adverse effects.

### Telomere shortening and dysfunction

Radiation-induced cellular senescence is closely associated with accelerated telomere shortening and subsequent dysfunction. Telomeres, repetitive nucleotide sequences at chromosome ends, maintain genomic integrity by preventing aberrant DNA damage signaling and chromosomal fusion. Ionizing radiation exacerbates telomere attrition by directly causing DNA strand breaks within telomeric regions 35. Due to their unique structure and the presence of shelterin complex components, telomeres are particularly inefficiently repaired 36. Persistent telomere dysfunction activates the DDR pathway—specifically the ATM/ATR signaling cascade—ultimately triggering the senescence program 37. This telomere-mediated senescence serves as a critical tumor-suppressive mechanism, preventing the proliferation of genomically unstable cells 38, 39. Furthermore, dysfunctional telomeres enhance the chronic secretion of SASP factors, significantly influencing tissue remodeling and inflammatory responses within the tumor microenvironment, thereby modulating tumor progression and treatment outcomes 40-42.

### Epigenetic alterations

Epigenetic modifications are key regulatory elements in radiation-induced cellular senescence. Radiotherapy significantly alters the epigenetic landscape through mechanisms including changes in DNA methylation, histone modifications, and chromatin remodeling 43. Alterations in DNA methylation patterns resulting from radiation stress often lead to sustained silencing or activation of genes involved in cell cycle arrest and senescence 44. Histone modifications—such as methylation and acetylation of lysine residues—regulate chromatin structure, influencing transcriptional accessibility and gene expression patterns, thereby reinforcing the senescent phenotype 45, 46. Additionally, chromatin remodeling complexes, such as SWI/SNF (SWItch/Sucrose Non-Fermentable), undergo regulatory changes following irradiation, further consolidating the transcriptional signature characteristic of senescent cells 47, 48 Collectively, these epigenetic alterations establish and maintain the senescent phenotype, reinforcing a durable cell-cycle exit (often stable under persistent damage signaling). In the context of radiotherapy, fractionated exposure may progressively reinforce these epigenetic barriers, thereby contributing to a durable transcriptional reprogramming that sustains both senescence-associated phenotypes and immunomodulatory signaling 44, 46. A deeper understanding of these epigenetic mechanisms may provide new therapeutic targets for modulating senescence and improving clinical outcomes following radiotherapy.

**Figure 1:**
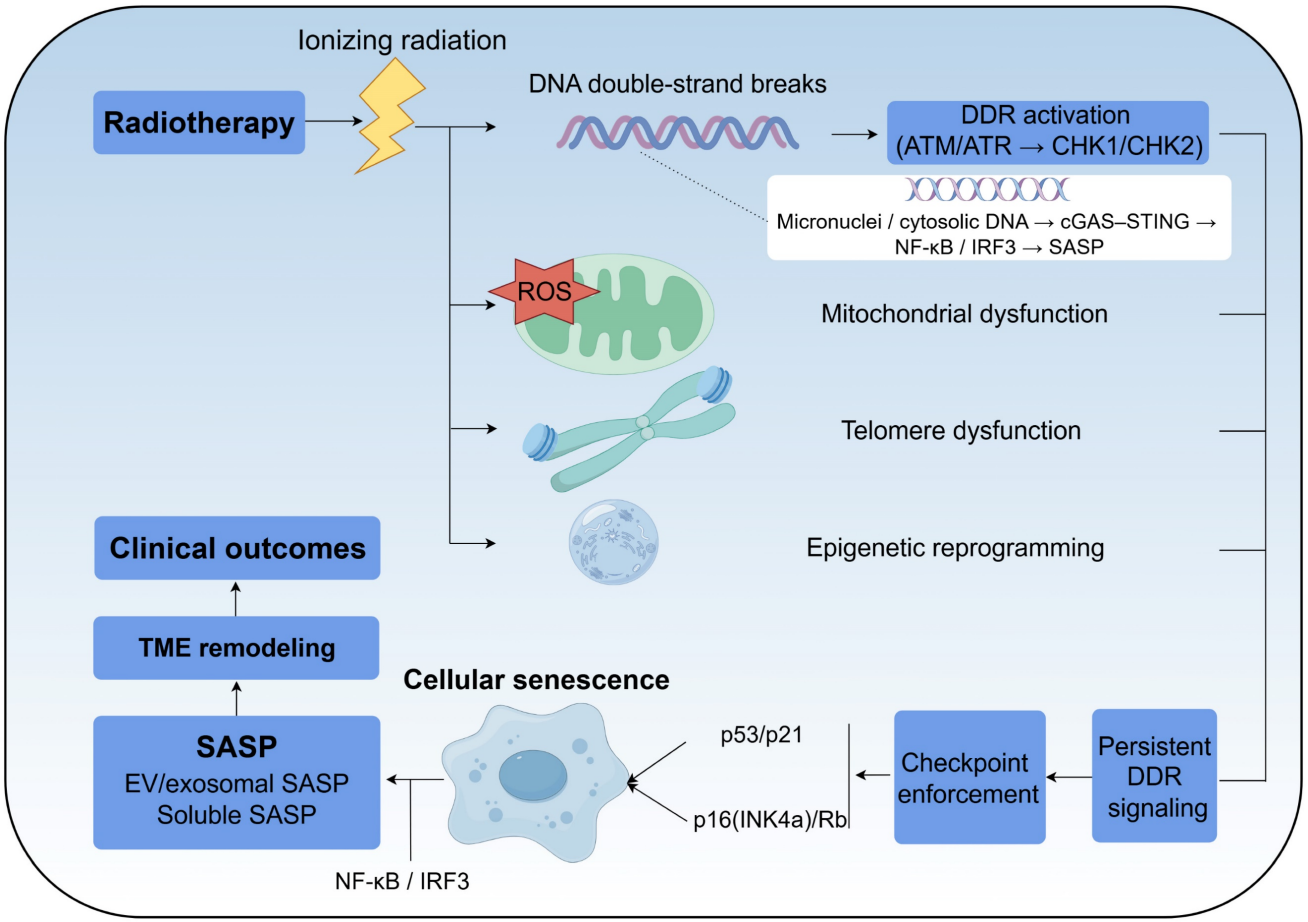
** Core pathways linking ionizing radiation to stable senescence and SASP induction.** Ionizing radiation induces DNA double-strand breaks and oxidative injury, triggering ATM/ATR-dependent DDR signaling. Persistent or irreparable damage engages the p53-p21 and p16(INK4a)-Rb axes to enforce durable cell-cycle arrest. ROS accumulation, telomere dysfunction and epigenetic remodeling reinforce this program. In parallel, micronuclei and cytosolic chromatin activate cGAS-STING, which signals through IRF3 and NF-κB to induce type I interferons and inflammatory SASP factors (such as IL-6, IL-8, chemokines and MMPs). These outputs drive paracrine remodeling of tumor and normal-tissue microenvironments in a time- and context-dependent manner.

Taken together, irradiation-induced DNA damage, ROS accumulation, telomere dysfunction, epigenetic remodeling, and cytosolic DNA sensing converge on the p53/p21 and p16(INK4a)-Rb axes to establish a typically stable senescent state and to shape the accompanying SASP programs. These interconnected pathways are summarised schematically in **Figure 1**.

### Cytosolic DNA sensing and cGAS-STING signaling as an upstream driver of SASP

Beyond nuclear DNA double-strand breaks, irradiation can produce micronuclei and cytoplasmic chromatin fragments via mitotic errors and defective genome maintenance 49. These aberrant DNA structures activate the cyclic GMP-AMP synthase (cGAS)-stimulator of interferon genes (STING) pathway, connecting radiation-induced genomic stress to inflammatory gene expression 50. STING activation subsequently signals through interferon regulatory factor 3 (IRF3) and NF-κB, inducing type I interferon responses and amplifying the expression of SASP components 51. Consequently, the cGAS-STING pathway acts as a mechanistic bridge that translates persistent DNA damage into sustained paracrine signaling.

From a radiotherapy perspective, this axis clarifies why senescence frequently involves a pro-inflammatory secretome instead of a quiescent arrest, and why the same program can aid immune surveillance shortly after treatment yet promote chronic inflammation and tissue remodeling if senescent cells persist. These insights encourage therapeutic approaches that either promote the timely clearance of senescent cells or dampen chronic SASP activity, depending on the specific disease context and treatment timeline.

## The impact of radiotherapy-induced cellular senescence on cancer treatment

Building on the upstream mechanisms described in Section 2, this section examines how dose delivery and cell-fate decisions shape whether radiotherapy-induced senescence contributes to durable tumor control or instead supports relapse and late toxicity.

Radiotherapy does not impose a single cellular fate. Depending on the dose, fractionation, and microenvironmental factors such as oxygenation, as well as intrinsic cellular checkpoints like p53 pathway integrity, irradiated cells may undergo clonogenic death, enter a stable senescent state, or recover and proliferate. Modern delivery techniques further influence these outcomes by altering the spatial and temporal distribution of the radiation dose. Consequently, interpreting radiotherapy-induced senescence requires a fate-centric perspective that distinguishes between its role as a tumor-suppressive endpoint and its function as a persistent, microenvironment-altering state linked to relapse and late toxicity.

### Radiotherapy modality and dose-delivery parameters shape senescence induction

Radiotherapy does not impose a single, uniform cellular fate. Following irradiation, tumor and stromal cells may undergo clonogenic death, durable senescence, or, in some contexts, partial recovery or escape. The balance among these outcomes depends not only on intrinsic cellular factors like DNA repair capacity and checkpoint integrity, but also on the spatial and temporal pattern of dose delivery. Consequently, modern radiotherapy modalities can differ from conventional photon therapy in their capacity to induce senescence, primarily by modulating dose heterogeneity, the extent of low-dose exposure to surrounding tissues, integral dose, linear energy transfer (LET) and relative biological effectiveness (RBE) patterns, and dose rate 2, 52-54.

Intensity-modulated radiotherapy (IMRT) and similar photon-based conformal techniques typically improve target conformity while distributing a broader "low-dose bath" to adjacent normal tissues. From a senescence perspective, this expands the volume of stromal and vascular compartments exposed to biologically significant low or intermediate doses. Even when such exposures fall below thresholds for overt cell death, they can promote persistent DNA damage signaling and long-lived senescent states, thereby increasing the overall senescent burden and senescence-associated secretory phenotype (SASP) footprint outside the primary tumor 55, 56.

Proton therapy often reduces the integral dose to normal tissues due to its finite range, which may correspondingly reduce senescence induction in uninvolved tissues and mitigate late toxicity. Simultaneously, protons exhibit variability in LET and, consequently, in biological effectiveness near the distal edge of the Bragg peak. The higher LET in this region can produce more complex DNA damage, potentially shifting the balance among cell death, stable senescence, and inflammatory signaling in a tissue- and context-dependent manner. These characteristics highlight why both physical dose and local radiation quality must be considered when evaluating senescence following proton irradiation 5, 35, 57.

Ultra-high-dose-rate FLASH radiotherapy introduces an additional temporal dimension. Preclinical studies indicate that FLASH can spare normal tissues while maintaining tumor control, with proposed mechanisms including radiation-chemical oxygen depletion and other dose-rate-dependent effects on radical kinetics and inflammatory cascades. From the perspective of senescence biology, these mechanisms are predicted to preferentially reduce chronic normal tissue injury and the accumulation of senescent cells, although the magnitude and generalizability of this effect likely depend on tissue oxygenation, fractionation, and the tumor microenvironment 4.

Collectively, these observations support a delivery-centric framework in which differences in modality and dose delivery reshape the burden and anatomical distribution of senescent cells and their SASP in both tumors and adjacent normal tissues. This reshaping, in turn, may influence durable tumor control, recurrence risk, and late normal tissue toxicity 55. Importantly, much of the current evidence remains preclinical or inferential; therefore, well-designed translational studies are required to quantify senescence biomarkers across modalities and to define clinically actionable "senescence-sparing" delivery principles 58, 59.

### Inhibition of tumor proliferation and cellular cycle arrest

Therapy-induced senescence is defined by durable cell-cycle arrest with preserved metabolic activity 58. In the setting of radiotherapy, this state can limit clonogenic expansion even when immediate cell death is incomplete, thereby contributing to short-term tumor control. Cell-cycle blockade is most commonly enforced through the p53/p21 axis and the p16(INK4a)/Rb axis, which converge on sustained repression of E2F-dependent proliferation programs. Depending on cellular context, senescent arrest may occur in G1 or G2 60, 61, and tumor cells frequently display a prominent G2/M arrest after irradiation 52. Radiation-induced cellular senescence is triggered by DNA damage, mitochondrial dysfunction causing ROS production and oxidative stress, and alterations in miRNA expression and epigenetic profiles 62, 63.

Importantly, no single marker is sufficient to define senescence *in vivo*. Robust identification typically requires a panel-based approach that integrates cell-cycle inhibitors (such as p21 and p16(INK4a)) 64, lysosomal features (enescence-associated β-galactosidase (SA-β-gal) activity) 65, and evidence of persistent damage signaling (for example, γH2AX foci), together with morphological and transcriptional changes. This multi-parameter definition is particularly relevant in radiotherapy studies, where transient arrest and stress responses can otherwise be mistaken for stable senescence.

### Senescence-associated secretory phenotype (SASP): inflammatory cytokines, chemokines, proteases, and growth factors

Senescence is characterized by the release of the SASP. SASP components are divided into two groups: soluble SASP (sSASP) and exosome/extracellular vesicle SASP (eSASP) 59. sSASP contains interleukins (IL-6 and IL-1α), inflammatory cytokines (C-X-C motif chemokine ligands and CC-chemokine ligands), growth factors (insulin-like growth factors), and proteases (matrix metalloproteinases and serine proteases), while eSASP includes miRNA and protein-carrying bioactive exosomes 66, 67. Importantly, the composition of the SASP is highly context-dependent, varying according to cell type, temporal stage, and radiotherapy regimen. For instance, in glioblastoma models, radiation-induced senescent astrocytes promote myeloid-cell recruitment and immunosuppressive remodeling 68, whereas senescent endothelial cells may shape immune surveillance programs differently 8. This heterogeneity likely explains why the SASP can restrain tumors in some contexts yet promote tumorigenesis in others.

The SASP factors are secreted by the senescence cells with the activation of various pathways 9, including the nuclear factor κB (NF-κB) 69, DNA damage response (DDR) 69, cyclic GMP-AMP synthase -stimulator of interferon genes (cGAS-STING) 70 and Janus kinase -signal transducer and activator of transcription (JAK-STAT) 71, etc. The SASP has the anti-tumorigenic function by reinforcement of the cell cycle arrest associated with senescence and recruitment of immune cells in TME. the SASP strengthens the permanence of senescence-associated cell cycle arrest rather than initiating it 72. SASPs attracts immune cells, like macrophages, natural killer (NK) cells, and T cells to mediate immune clearance of senescent cancer cells, leading to impedance of tumor progression and tumor regression 56, 69, 73, 74. Senescent prostate cancer (PCa) cells can drive the transformation of macrophages from the tumor-promoting M2 phenotype to the antitumor M1 phenotype via the SASP 75. The impact of the SASP varies significantly based on the context and cell type, and it can change throughout the different stages of cancer progression 9. With the time going, cellular senescence creates a tumor promoting environment through a secretion of SASP 53, promoting epithelial-mesenchymal transition (EMT) initiation 76, cellular plasticity induction 77, angiogenesis 78, tumor invasion 79, 80, activation of fibroblasts 81.

### Impact on tumor recurrence and drug resistance

Therapeutic resistance in cancer occurs when tumors continue to grow despite initially responding to treatment, leading to poor prognoses and preventing clinical success. While cellular senescence can initially suppress tumor growth as the cell tries to fix the damaged DNA, it also considered to be an important cause of tumor promoting and radiotherapy resistance. Notably, senescence is not an irreversible endpoint. Therapy-induced senescent tumor cells can escape this state and acquire enhanced plasticity or stem-like features, a process linked to disease recurrence and treatment resistance 82. This connection provides a rationale for post-radiotherapy interventions that target senescent cells, such as senolytics or immune-mediated clearance, to prevent their persistence and the outgrowth of resistant variants.

Radiation can cause a temporary halt in cell growth, which is often followed by a phase of proliferative recovery. This recovery phase has the potential to contribute to disease recurrence by the pro-tumorigenic environment induced by SASP 83. Recent research has found that SASP factors have these roles by promoting the dedifferentiation of cancer cells. These factors have the ability to enhance cellular reprogramming and trigger the expression of stem cell markers in nearby cells, leading to a more aggressive and treatment-resistant cancer phenotype 84, 85. Understanding the balance between the beneficial and detrimental effects of SASP is crucial for improving long-term cancer therapy outcomes. Beyond the role of SASP, studies have observed that senescent brain cells contribute to tumor recurrence in glioblastoma by promoting the peripheral recruitment of myeloid inflammatory cells 68, 86. A recent study demonstrates that removing radiation-induced senescent astrocytes can decrease the recurrence of radioresistant malignant glioma in the brain 68, 87. Initial senescence of tumor cells caused by radiation, along with the NF-κB-dependent release of acute SASP cytokines, has been identified as a potential pharmacological target to address radiotherapy failure 88.

### Effects on surrounding normal and stromal cells

**Figure 2:**
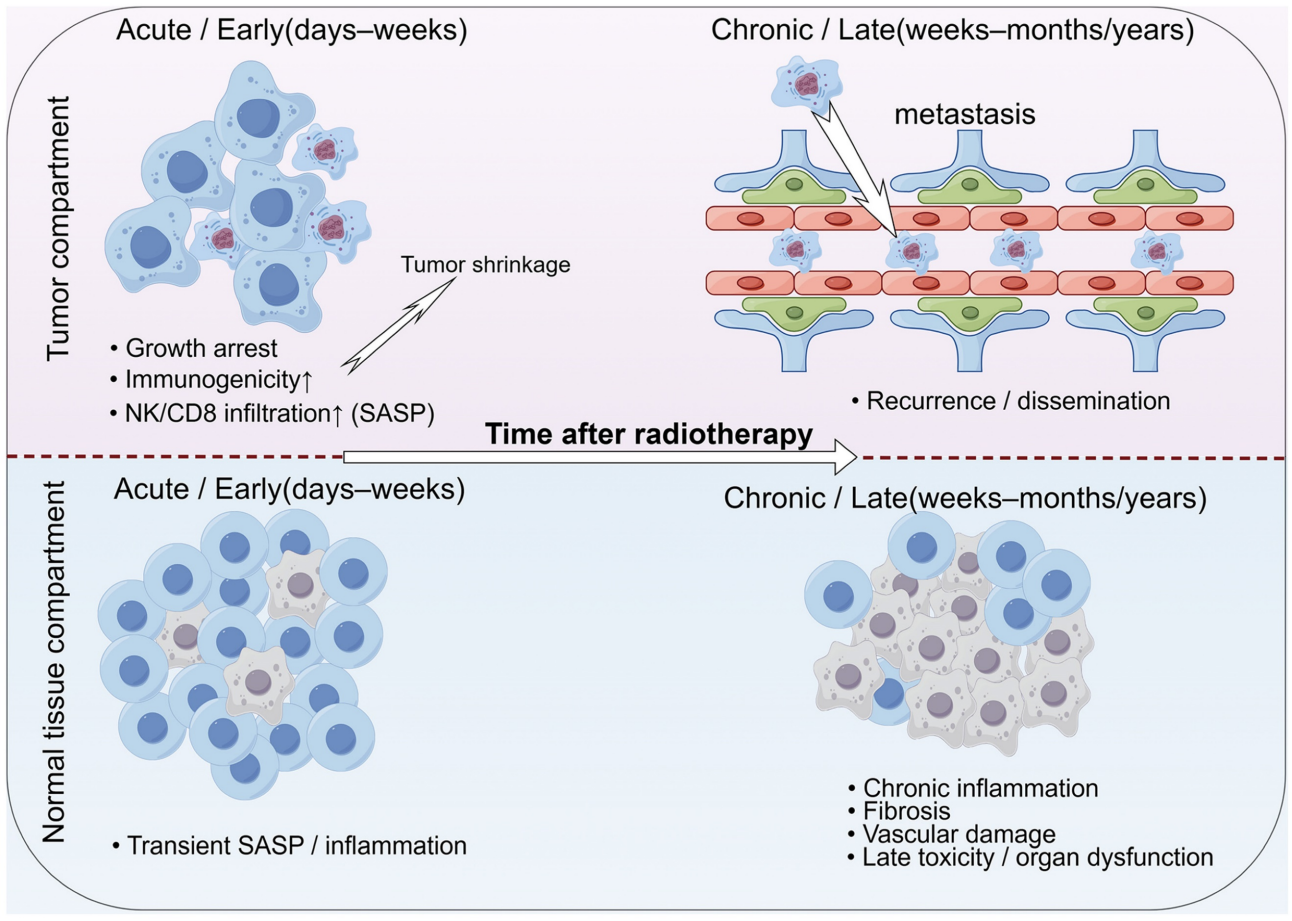
** Radiotherapy-induced cellular senescence as a double-edged, time-dependent process.** Shortly after radiotherapy, senescence in tumor cells can contribute to durable growth arrest and enhance anti-tumor immune responses, supporting tumor control. Over time, however, persistent senescent cells and chronic SASP in both tumor and normal tissues can drive epithelial-mesenchymal transition (EMT), cancer cell plasticity, immune suppression, fibrosis and vascular dysfunction, thereby promoting tumor recurrence, metastasis and late normal tissue toxicity.

Beyond cancer cells, cancer tissue includes stromal cells that form the tumor microenvironment. Stromal cells with senescence are frequently observed 89, and treating tumors with radiotherapy can cause senescence in surrounding healthy tissue by the communication of with irradiated cells by SASP factors 90, 91. Among these stromal cells, fibroblasts, or cancer-associated fibroblasts (CAFs), are particularly prevalent and significantly influence various aspects of radiotherapy 55. Senescent stromal cells induced by radiotherapy can promote the growth of tumor cells, prevent cancer cells from radiation-induced death through paracrine signaling by cytokines 92, 93. SASP factors secreted by senescent stromal cells can stimulate the proliferation of nearby tumor cells 94. Not only do senescent tumor cells help evade the immune system and result in tumor recurrence, but the accumulation of senescent stromal cells also creates a chronically inflamed TME that protects early tumor cells 95. Collectively, these observations support the notion that radiotherapy-induced senescence exerts a context- and time-dependent influence on cancer therapy. In the early phase after treatment, senescence in tumor cells can contribute to durable growth arrest and enhance anti-tumor immunity, whereas persistent senescent cells and chronic SASP in both tumor and normal tissues progressively promote tumor recurrence, metastasis and late normal tissue toxicity. This double-edged, temporally dynamic role of radiotherapy-induced senescence is illustrated in **Figure 2**.

### Acute versus chronic senescence: temporal window defines outcomes

Clinically, the consequences of radiotherapy-induced senescence depend on whether senescent cells are transient and efficiently cleared (acute phase) or persist and accumulate (chronic phase). In the acute window, typically days to weeks after irradiation, p53/p21- and/or p16(INK4a)/Rb-mediated arrest suppresses clonogenic expansion, while an early, immunostimulatory SASP enriched with interferon and chemokine programs facilitates immune recruitment and senescence surveillance by NK cells and T cells 8, 73, 96.

In contrast, when senescent cells persist for weeks to months, sustained NF-κB and cGAS-STING signaling maintains a chronic SASP—characterized by factors such as IL-6, IL-8, TGF-β, and matrix-remodeling proteases—that remodels the extracellular matrix, promotes angiogenesis and cellular plasticity, and progressively skews the immune microenvironment toward myeloid-driven suppression and T-cell dysfunction 55, 56, 70, 77, 95. For example, in the irradiated brain, radiotherapy-induced astrocyte senescence has been shown to promote an immunosuppressive niche that facilitates glioblastoma regrowth, and clearing these senescent astrocytes mitigates recurrence 68, 87.

This temporal framework offers a practical design principle for combination therapy: early immunogenic priming should be preserved, while senolytics or senomorphics can be considered once a stable senescent burden and chronic SASP signatures are detected via longitudinal biomarkers and functional endpoints 53.

## Impact of radiotherapy-induced cellular senescence on the tumor microenvironment

Having outlined the treatment consequences of senescence in Section 3, this section focuses on how senescent tumor and stromal cells reshape the tumor microenvironment through the SASP.

**Figure 3:**
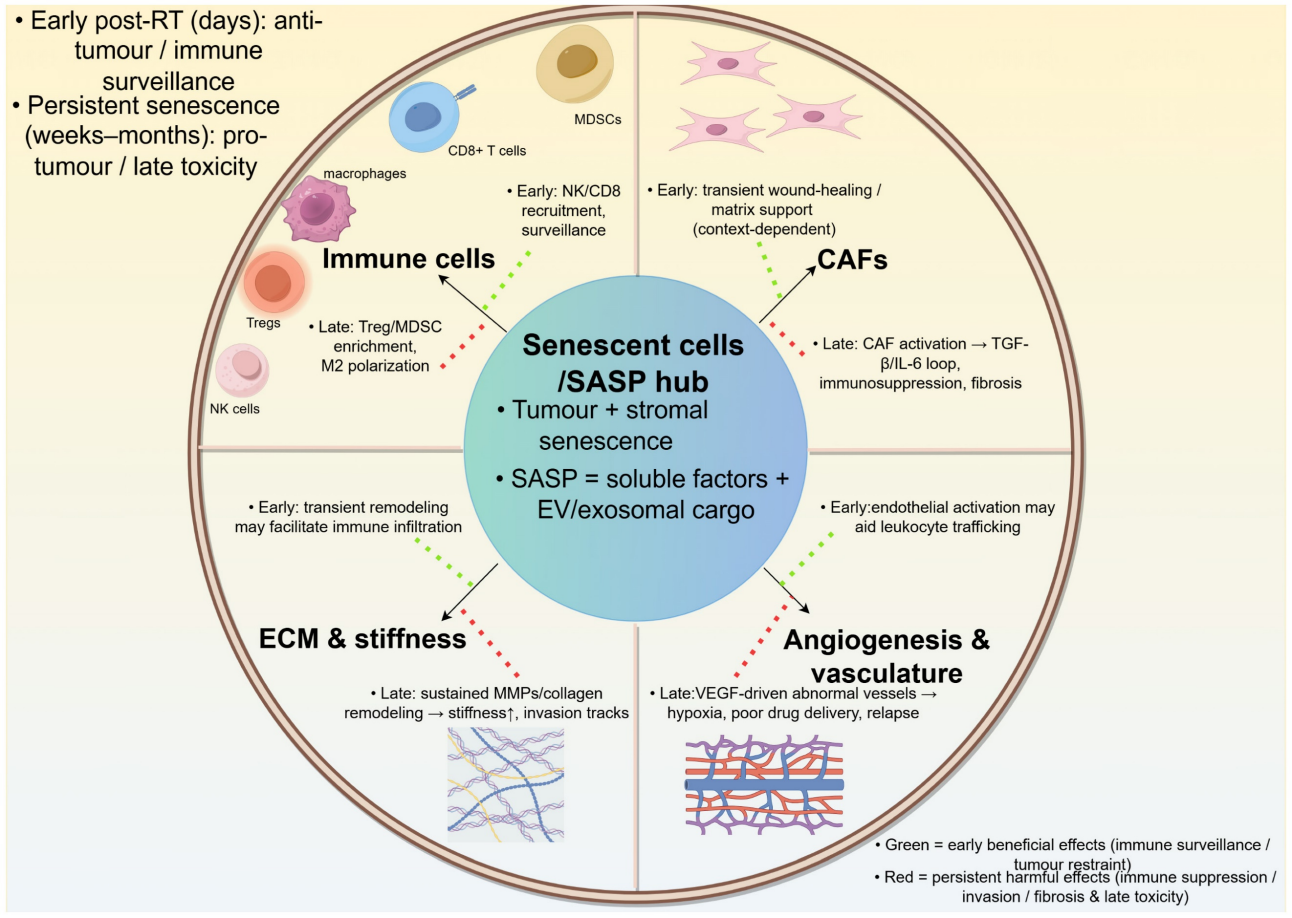
** SASP-driven remodeling of the tumor microenvironment after radiotherapy-induced senescence.** Senescent tumor and stromal cells secrete soluble and extracellular vesicle-associated SASP mediators that reshape extracellular matrix, activate fibroblasts/CAFs, remodel vasculature, and reprogram immune infiltration and function. In the revised schematic, green arrows denote early/anti-tumor consequences (immune recruitment and senescence surveillance), whereas red arrows denote persistent/pro-tumor consequences (chronic inflammation, CAF activation, angiogenesis, T-cell dysfunction, immune suppression, invasion and recurrence). The net effect depends on senescent-cell persistence, tissue context and treatment timeline.

Radiotherapy-induced senescent cells remodel the tumor microenvironment primarily through the SASP, which integrates signals from tumor and stromal compartments. SASP-derived cytokines, chemokines, growth factors and proteases dynamically influence the extracellular matrix, immune infiltrates, cancer-associated fibroblasts and tumor vasculature. This central “secretory hub” linking senescent cells to tumor microenvironment remodeling is summarised in **Figure 3**.

### Remodeling of the extracellular matrix

Radiotherapy-induced cellular senescence significantly remodels the tumor microenvironment through the regulation of the extracellular matrix (ECM) 97, 98. Senescent cells actively produce and secrete various matrix-modifying enzymes, such as matrix metalloproteinases (MMPs), collagenases, and elastases, leading to substantial alterations in the composition and structure of the ECM 99. The enzymatic activities of these proteins degrade ECM components such as including collagen, elastin, and fibronectin, thereby modifying the biomechanical properties and stiffness of the tissue. Increased ECM stiffness is a hallmark feature of the senescent microenvironment and can activate mechanosignaling pathways involved in tumor progression, cell migration, and metastatic dissemination 100, 101. Furthermore, ECM remodeling promotes the recruitment of immune cells and enhances interactions between tumor cells and surrounding stromal cells, fostering an inflammatory microenvironment conducive to cancer progression 102 Overall, radiotherapy-induced senescence-driven ECM alterations have profound implications for tumor aggressiveness, treatment resistance, and overall disease progression.

### Immune cell infiltration and activity

Radiotherapy-induced senescent cells primarily modulate immune responses within the tumor microenvironment via the SASP 103, 104. SASP factors include pro-inflammatory cytokines such as IL-6 (interleukin-6), IL-8, TNF-α, chemokinic inducers such as CCL2 and CXCL1, and growth factors such as granulocyte-macrophage colony-stimulating factor, GM-CSF, which together regulate the recruitment and polarization of immune cells 105. They promote the infiltration and activation of immune cells such as macrophages, neutrophils, T cells, and natural killer (NK) cells 106. However, the sustained presence of SASP may paradoxically foster an immunosuppressive environment characterized by an increase in regulatory T cells, myeloid-derived suppressor cells (MDSCs), dysfunctional/senescent CD8+ T cells 107 and immunosuppressive macrophages (M2 phenotype) 108. Senescent cells initially activate immune monitoring and ultimately promote long-term immune evasion, a dual capability that highlights their complex role in immune dynamics post-radiotherapy, ultimately influencing treatment outcomes and patient outcomes.

In preclinical glioblastoma models, cranial radiotherapy (RT) preferentially induces senescence in astrocytes. These senescent astrocytes subsequently drive peripheral myeloid recruitment and immunosuppressive remodeling via their senescence-associated secretory phenotype (SASP) chemokine programs. This process ultimately facilitates tumor regrowth following radiotherapy 68.

### Interactions with cancer-associated fibroblasts (CAFs)

Cancer-associated fibroblasts (CAFs) are key components of the tumor stroma and actively participate in tumor progression, invasion, and metastasis. Radiotherapy-induced senescent cells directly influence CAF function and behavior through SASP secretion 92, 93. SASP factors—particularly transforming growth factor-β (TGF-β) and interleukins such as IL-6 and IL-1β—drive fibroblast activation into CAFs and promote their differentiation and proliferation 109, 110. Activated CAFs further secrete ECM-remodeling enzymes, angiogenic factors, and inflammatory cytokines, creating a self-sustaining pro-tumorigenic stromal niche 111. This dynamic interaction significantly enhances the invasive capacity of tumor cells, promotes treatment resistance, and accelerates metastatic spread. Thus, the reciprocal crosstalk between senescent cells and CAFs constitutes a critical axis influencing tumor progression and therapeutic response.

### Angiogenesis and vascular remodeling

The SASP of radiotherapy-induced senescent cells includes potent angiogenic mediators such as vascular endothelial growth factor (VEGF), fibroblast growth factor (FGF), and IL-8 112. These molecules drive angiogenesis by stimulating endothelial cell proliferation, migration, and differentiation into new blood vessels 113. Additionally, SASP-induced angiogenic signaling leads to vascular remodeling, characterized by increased permeability, abnormal branching, and hemodynamic disturbances 74. Such aberrant vascular structures can enhance nutrient and oxygen supply to the tumor, accelerating tumor growth and facilitating metastasis 74. Altered vasculature also impairs the efficacy of subsequent treatments by reducing drug delivery efficiency and creating hypoxic regions, further exacerbating tumor progression and treatment resistance 114-116. Elucidating the mechanisms underlying senescence-induced angiogenesis and remodeling may provide novel therapeutic strategies to improve radiotherapy outcomes and suppress tumor aggressiveness. Taken together, accumulating evidence indicates that radiotherapy-induced cellular senescence in both tumor and stromal compartments profoundly remodels the tumor microenvironment by altering the extracellular matrix, immune contexture, fibroblast activation and vascular architecture. Representative preclinical and translational studies illustrating these context-dependent effects are summarised in **Table 1**, highlighting how the location and composition of the senescent cell population shape treatment response and disease course.

## Modulating the treatment strategy of radiotherapy-induced cell senescence

**Figure 4:**
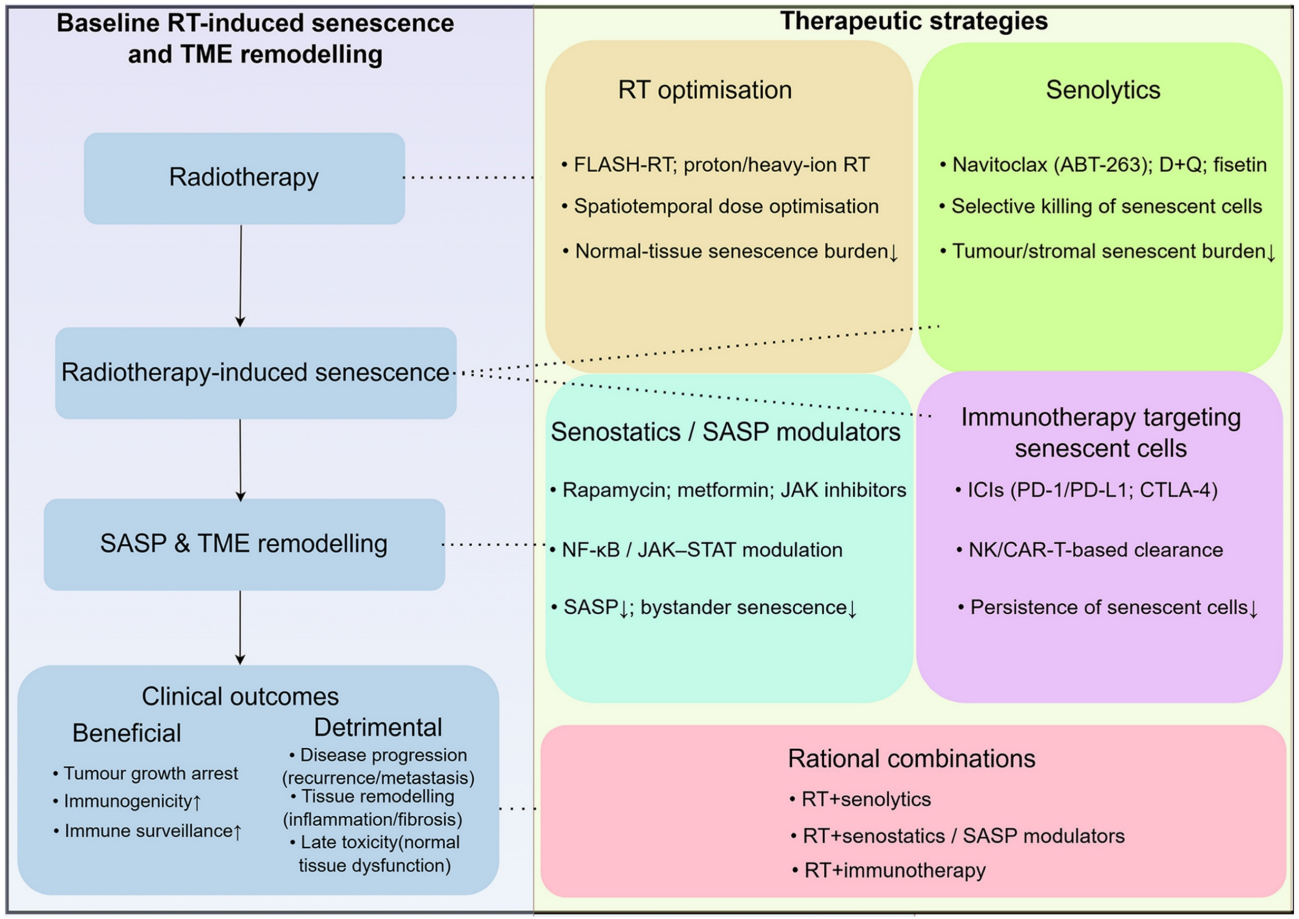
** Therapeutic strategies to modulate radiotherapy-induced senescence and SASP.** Radiotherapy induces senescence in tumor and stromal cells, leading to SASP production and TME remodeling, with mixed effects on tumor control, recurrence and normal tissue toxicity. Senolytics aim to selectively eliminate senescent cells, whereas senostatics and SASP-targeting agents seek to reprogramme or dampen harmful secretory profiles. Immunotherapeutic approaches can enhance immune-mediated clearance of senescent cells. Optimisation of radiotherapy itself, for example through FLASH-RT or proton therapy, may preferentially reduce senescence in normal tissues while preserving anti-tumor senescence. Rational combinations of these strategies offer opportunities to tilt the balance toward durable tumor control with reduced long-term toxicity.

The key therapeutic challenge after radiotherapy is to harness beneficial early senescence while preventing persistent senescent cells and chronic SASP from driving relapse and late toxicity. Conceptually, these approaches can be grouped into four major categories: (i) senolytics that selectively eliminate senescent cells, (ii) senostatics and SASP modulators that reprogramme or dampen harmful secretory profiles, (iii) immunotherapeutic strategies that enhance immune-mediated clearance of senescent cells, and (iv) optimisation of radiotherapy delivery itself to spare normal tissues while preserving anti-tumor senescence. These interlocking strategies and their relationships to radiotherapy-induced senescence and SASP are summarised in **Figure 4**, and key representative agents are further detailed in** Table 2**. Because radiotherapy-induced senescence is heterogeneous across compartments and evolves over time, effective combinations must prioritize temporal and biological selectivity over indiscriminate senolysis or SASP blockade. We therefore advocate for a phase-aware scheduling strategy, typically administered post-RT when persistent senescence dominates, combined with biomarker-guided adaptation. This approach aims to preserve the early antitumor benefits of therapy while limiting chronic SASP-driven immunosuppression, fibrosis, and relapse.

### Mechanisms and examples of drugs (Senolytics) that remove senescent cells, clinical trials, and effects

In radiotherapy, senolytics are primarily investigated to mitigate the long-term accumulation of senescent cells, which drive chronic SASP signaling, fibrosis, and tissue dysfunction. Most supporting evidence is still preclinical, and a key translational challenge involves defining optimal timing and selectivity. This is necessary to ensure that clearing harmful senescent stromal cells does not interfere with beneficial early antitumor responses.

Drugs that selectively kill senescent cells, termed senolytics, have proved beneficial in animal models of many age-associated diseases 117. Therapeutics for killing senescent cells could take the form of senoptotic or senolytic small molecules or immune-based clearance 118. Recent studies on senolytics have highlighted their potential in targeting senescent cells to treat age-related diseases. Dasatinib and quercetin have been shown to reduce senescent cell burden and improve physical function in aged individuals, demonstrating potential in treating frailty and enhancing overall healthspan 119. Fisetin, a natural flavonoid, has been found to decrease senescent cells and extend healthspan and lifespan in preclinical animal models, highlighting its promise as a therapeutic agent for age-related diseases 120. Navitoclax (ABT263), originally developed for cancer therapy, has demonstrated significant efficacy in clearing senescent cells and rejuvenating aged hematopoietic stem cells, thereby improving tissue regeneration and function 121.

### Drugs that inhibit aging pathways (Senostatics)

Compared with senolytics, senomorphics and senostatics may be particularly attractive following radiotherapy, as they aim to attenuate chronic SASP outputs without necessarily eliminating senescent cells. This approach could offer a wider therapeutic window in normal tissues, although its durable efficacy and on-target safety remain key open questions. In contrast to senolytics, senostatics do not kill senescent cells but inhibit paracrine signalling and thus block the 'proliferation' of senescence due to the bystander effect 122.

Senostatics exert their effects by modulating pathways associated with cellular senescence, primarily through the suppression of the senescence-associated secretory phenotype (SASP), control of cell cycle checkpoints, antioxidant activity, and metabolic pathway adjustments. These agents aim to mitigate the deleterious impact of senescent cells on tissues and organs, thereby attenuating the progression of age-related diseases 123. Senostatics, including agents such as rapamycin, metformin, and JAK inhibitors, exhibit significant potential in mitigating age-related diseases. Recent clinical trials on senostatics have demonstrated their potential in ameliorating age-related diseases through various mechanisms. Rapamycin has been shown to enhance immune function and reduce infection rates in elderly populations, highlighting its role in modulating mTOR signaling pathways 124, 125. Metformin improves metabolic health and reduces systemic inflammation in older adults by regulating metabolic and nonmetabolic pathways in skeletal muscle and adipose tissues 126. JAK-STAT inhibition is a representative senomorphic strategy supported primarily by preclinical evidence to suppress core SASP transcriptional programs; its clinical translation as a senescence-targeted approach in RT contexts remains preliminary 119.

### Targeting the SASP: anti-inflammatory drugs, cytokines, and chemokine inhibitors

SASP modulation aligns conceptually with radiotherapy toxicities, which are driven by chronic inflammation, matrix remodeling, and pro-fibrotic signaling. Consequently, anti-inflammatory and cytokine-targeted strategies are increasingly considered as adjuncts to radiotherapy, particularly for mitigating late effects like fibrosis.

Some senescent cells acquire a senescence-associated secretory phenotype (SASP), characterized by the secretion of various factors. These include inflammatory cytokines like TNFα, IL-6, and IL-8, which induce apoptosis and insulin resistance; chemokines that attract and activate immune cells; matrix metalloproteinases (MMPs) such as MMP-3, -9, and -12, leading to tissue destruction; TGFβ family members, activins, and inhibins, contributing to fibrosis and stem cell dysfunction; serpines like PAI-1 and -2, promoting blood clotting and fibrosis; growth factors that can enhance tumor spread; bioactive lipids such as bradykinins, ceramides, and prostaglandins, causing inflammation and tissue dysfunction; and microRNAs (miRNAs) and exosomes, which carry cytotoxic and senescence-inducing cargos, affecting stem cells, inflammation, and insulin resistance 127-130.

### Enhance the immune system and remove senescent cells: immunotherapy methods, vaccines against senescent cells

Because senescence can recruit immune effectors shortly after irradiation but promote immune dysfunction when it persists, the efficacy of immunotherapy to clear senescent cells likely depends on treatment timing. Consequently, translational studies should combine immunotherapeutic strategies with longitudinal senescence biomarkers to prevent the impairment of early antitumor immunity.

Recent advancements in immunotherapy have focused on enhancing the clearance of senescent cells to mitigate age-related pathologies. Immune checkpoint inhibitors, like anti-PD-1 and anti-CTLA-4 antibodies, boost T cell activity to eliminate senescent cells, showing reduced inflammation and improved physical function in early trials 96, 131-133. CAR-T cell therapy engineers T cells to target senescent cells, with promising results in initial trials 134. NK cell-based therapy enhances natural killer cell activity, effectively targeting and reducing senescent cells and inflammation 96, 135. Combining immune therapies with senolytic agents like dasatinib and quercetin sensitizes senescent cells to immune clearance, improving outcomes in patients with diabetic kidney disease 136, 137. Vaccines targeting senescent cells aim to stimulate the immune system to recognize and eliminate these cells, thereby mitigating age-related pathologies 131, 138. These vaccines introduce antigens specific to senescent cells, prompting the immune system to produce antibodies and activate cytotoxic T cells against these targets 134. By using senescence-associated surface proteins as antigens, the immune-mediated clearance of senescent cells can be achieved, reducing inflammation and improving tissue function 136. In one study, a vaccine targeting the p16(INK4a) protein demonstrated significant reduction in senescent cell markers and improved physical performance in treated mice 131. Another clinical trial involving a vaccine directed against senescence-associated β-galactosidase showed decreased levels of pro-inflammatory cytokines in elderly participants 134. These findings highlight the potential of senescence-targeting vaccines to alleviate age-related diseases and enhance healthspan 136, 137.

### Combination therapy with radiotherapy: synergistic effects with chemotherapy, combination of targeted therapy

Combining chemotherapy or targeted therapy with radiotherapy enhances the clearance of senescent cells by exploiting their increased susceptibility to DNA damage and stress 96. For instance, doxorubicin, a chemotherapeutic agent, increases the immunogenicity of senescent cells, making them more recognizable to the immune system, and when combined with radiotherapy, leads to increased apoptosis of senescent cells 131, 134, 139. Similarly, targeted therapies such as BCL-2 inhibitors increase the vulnerability of senescent cells to apoptosis, and when used with radiotherapy, they result in higher rates of senescent cell death, as demonstrated in studies combining venetoclax with radiotherapy 136. Clinical trials for these combined treatments have shown promising results, including reduced inflammation and improved tissue regeneration, highlighting their potential to enhance therapeutic efficacy and improve patient outcomes 137, 140.

Radiotherapy synergizes with immunotherapy by enhancing antigen release, promoting innate immune activation, and reshaping the immune microenvironment 141. Senescence introduces a schedule-dependent dimension to this interaction. Shortly after radiotherapy, senescent tumor cells and damaged stromal compartments can produce an acute inflammatory secretory program that facilitates immune cell recruitment and immune-mediated clearance, potentially amplifying responses to immune checkpoint blockade 142. Conversely, the persistence of senescent cells and chronic SASP signaling may foster immune dysfunction through myeloid skewing, regulatory T-cell enrichment, and impaired cytotoxic activity, thereby promoting immune escape and relapse 143. These dynamics indicate that the benefit of combining radiotherapy with immunotherapy is likely highly dependent on treatment timing and patient-specific factors. A rational strategy to improve durable tumor control would leverage this early immune activation while limiting prolonged SASP-driven immunosuppression. The optimal sequencing, biomarkers for patient selection, and safety boundaries, however, remain undefined, underscoring the need for translational studies that longitudinally track senescence and immune readouts following combined treatment.

To facilitate comparison across modalities and to highlight their mechanisms and RT-related implications, the main senescence-targeted strategies discussed in this section are ummarized in **Table 2**. These distinct mechanistic classes of intervention raise a final translational question: how to deploy senescence-directed strategies in patients using clinically tractable biomarkers, trial evidence, and safety-aware scheduling. This question is addressed in the following section.

## Clinical significance and future research directions

This section highlights the translational landscape—ongoing trials, biomarker strategies, personalized implementation, and long-term safety—needed to move senescence-targeted approaches toward routine clinical radiotherapy.

### Current clinical trials and research progress

From a translational perspective, current support for senescence-targeting interventions rests on two distinct lines of evidence: (i) early-phase human trials of senotherapeutics, conducted largely outside of radiotherapy, which demonstrate feasibility and preliminary safety; and (ii) applications adjacent to radiotherapy where the mechanistic rationale and efficacy data are primarily derived from preclinical models. Here, we summarize representative human studies as a translational foundation, while explicitly distinguishing them from the specific evidence gaps that remain within radiotherapy itself.

Senolytics, a promising class of drugs designed to selectively eliminate senescent cells, are being explored through various pharmacological strategies, including small molecules, peptides, and antibodies 122, 123, 127, 136, 144. Known for their resistance to apoptosis, senescent cells can be targeted by senolytic agents that vary in specificity towards different cell types and tissues. To date, five to six distinct signaling pathways have been identified for drug development, including Bcl-2, PI3K/AKT/mTOR, HIF-alpha pathways, TK inhibitors, and HSP-90 inhibitors 144. Current clinical trials focusing on the SASP are investigating its role in various age-related diseases and potential therapeutic interventions. For instance, a study conducted by the University of Turin is examining SASP biomarkers in gingival crevicular fluid to assess their impact on periodontal regeneration outcomes 145. For example, the senolytic combination dasatinib plus quercetin (D+Q) has advanced into early-phase clinical testing, including a randomized pilot trial in idiopathic pulmonary fibrosis (ClinicalTrials.gov: NCT02874989) 119, which provides feasibility and adverse-event profiles that can inform scheduling and safety considerations for radiotherapy-adjacent translation.

Radiotherapy-induced cellular senescence plays a dual role in cancer therapy, both inhibiting and potentially promoting tumor growth. Recent studies reveal that radiotherapy induces cellular senescence, leading to reduced tumor growth, as seen in breast cancer models 146, 147. Senescent cells release cytokines such as IL-6 and IL-8, enhancing anti-tumor immune responses 146, 148. However, senescent cells can alter the tumor microenvironment, promoting tumor recurrence and metastasis 149-151. In lung cancer models, radiotherapy-induced senescence increased pro-inflammatory cytokines, correlating with tumor recurrence 148. In prostate cancer, senescent cells caused neighboring healthy cells to enter senescence, potentially leading to recurrence and resistance 151. Many preclinical radiotherapy models have used the anti-aging drug Navitoclax for radiotherapy, showing potential to reduce side effects and delay recurrence 87, 152, 153. In parallel, navitoclax (ABT-263), a BCL-2/BCL-xL inhibitor commonly employed as a senolytic tool compound in preclinical radiotherapy research, has been assessed in early-phase oncology trials (ClinicalTrials.gov: NCT00481091). These trials identified thrombocytopenia as a principal dose-limiting toxicity, underscoring a major translational challenge for radiotherapy combination approaches. In melanoma, combining radiotherapy with PD-1 inhibitors has enhanced anti-tumor effects 57, 154. Therefore, understanding the mechanisms of radiotherapy-induced senescence is crucial. For instance, radiotherapy induces senescence through the ATM and p53 pathways, offering new therapeutic targets 155-157, research on SASP factors will further optimize radiotherapy strategies 87, 158, 159.

Looking forward, the key near-term priorities involve trial design rather than drug discovery alone. These include defining an actionable therapeutic window, such as early post-radiotherapy intervention versus late treatment for established toxicity or relapse. Another priority is selecting endpoints that capture both tumor control and late tissue injury. A further consideration is implementing patient stratification using circulating and tissue-based senescence biomarkers. These steps are essential to translate senescence-targeted combinations from proof-of-concept models into clinically testable protocols.

### Biomarkers for aging detection

Cellular senescence is characterized by a state of permanent cell cycle arrest, elevated activity of SA-β-gal, resistance to apoptotic stimuli, and deregulated metabolism 160. Morphologically, senescent cells exhibit notable changes: they become flattened, and their nuclei enlarge, signifying essential hallmarks of this state 161. Recent advances have identified several key biomarkers for detecting cellular senescence. One critical marker is SA-β-gal, a lysosomal enzyme detectable at pH 6 in senescent cells' lysosomes 65, 162. SA-β-gal was the first marker identified for cellular senescence, as its activity significantly increases in nearly all senescent cells, and it is now used as a specific marker of cancer cell senescence 65. Another hallmark of senescence is persistent cell cycle arrest, regulated by cyclin-dependent kinase (CDK) inhibitors such as p16(INK4a) (CDKN2A), p27Kip1 (CDKN1B), and p21Waf1 (CDKN1A, CIP1) 163. p16(INK4a) is particularly notable due to its accumulation in aging tissues and senescent cells 164. Additionally, Rb protein, when hypophosphorylated, binds to the E2F family transcription factors, inhibiting their activity and causing cell cycle arrest 164. Senescence-associated heterochromatin foci (SAHF) further contribute to the stable silencing of proliferation-promoting genes, making the level of phosphorylated Rb (p-Rb) another crucial marker of cellular senescence 164. The senescence-associated secretory phenotype (SASP), characterized by pro-inflammatory cytokines such as IL-6 and IL-8, highlights the complex role of senescent cells in aging and disease 165, 166. SASP, driven by persistent DNA damage response (DDR), stands out as a significant marker of cellular senescence 29, 167. Moreover, biomarkers such as the downregulation of lamin B1, and the increased expression of p19ARF, γ-H2AX, and PAI-1, are also indicative of senescence 168. γ-H2AX foci, indicating persistent DNA damage, are prevalent in senescent cells 169. Emerging markers like HMGB1, which translocates from the nucleus to the cytoplasm in senescent cells, are gaining attention as well 170. Therefore, detecting multiple indicators is essential to confirm the occurrence of cellular senescence.

Practical translation requires senescence biomarkers that are measurable in patients rather than solely in experimental models. In radiotherapy, candidate readouts include tissue-based markers such as p16(INK4a)/p21 induction, reduced Ki-67, hypophosphorylated Rb, and persistent DNA damage response foci like γH2AX/53BP1 and telomere dysfunction-induced foci. Additional hallmarks encompass lysosomal and metabolic changes, evidenced by SA-β-gal activity 62 and lipofuscin accumulation, along with circulating signatures that reflect systemic or local SASP output, including IL-6/IL-8, CCL2, TGF-β, and PAI-1 63, 160. Detectable in plasma, extracellular vesicle-associated “eSASP” cargo, such as specific miRNAs and proteins, also serves as a potential biomarker source. Emerging imaging approaches, including activity-based probes or radiotracers designed to report senescence-associated enzymatic activity or surface-marker expression, provide a route to noninvasive, spatially resolved assessment, though these methods currently lack standardization for routine clinical use.

Most individual markers are context-dependent and lack absolute specificity, complicating their interpretation following radiotherapy. For instance, p16(INK4a) and p21 can be induced by transient stress responses, while SA-β-gal assays are sensitive to tissue handling and sampling conditions. Circulating SASP factors are frequently confounded by baseline inflammation, infections, comorbidities, and concurrent therapies. Senescence is also spatially heterogeneous, differing between the tumor core and margin or in adjacent normal tissues, and temporally dynamic across acute and chronic phases, which renders single time-point measurements prone to misclassification. Consequently, clinically actionable assessment will likely require multi-analyte panels that combine complementary tissue and blood-based markers, ideally obtained longitudinally at pre-radiotherapy baseline, early post-radiotherapy, and late follow-up time points. Such panels would map senescent-cell burden and SASP trajectories to better inform the timing of senescence-targeted interventions.

### Personalized approach to medicine

Personalized therapies for inducing cellular senescence employ various strategies to selectively drive cancer cells into a state of senescence, enhancing therapeutic efficacy while minimizing side effects 171. Firstly, inducing DNA damage through specific chemotherapy drugs or radiation therapy, such as doxorubicin, effectively triggers senescence in tumor cells 54, 172-174. Secondly, activating tumor suppressor pathways, like the p53 pathway via MDM2 inhibitors, significantly increases the rate of senescence in cancer cells 175. Additionally, inducing oxidative stress through drugs like cisplatin raises the oxidative stress levels in tumor cells, promoting senescence 176, 177. Metabolic reprogramming also plays a crucial role; using glycolysis inhibitors or lipid metabolism modulators alters the metabolic state of cells to induce senescence 178. Combination therapies, such as using BCL-2 inhibitors alongside radiation therapy, can significantly enhance the clearance of senescent cells 152. Immunotherapy approaches, including immune checkpoint inhibitors (like PD-1 inhibitors) or CAR-T cell therapy, further improve the immune system's ability to target and clear senescent cells 179-181. Additionally, personalized monitoring of biomarkers, such as SA-β-gal and p16(INK4a), allows real-time assessment of treatment efficacy and adjustments to the therapeutic strategy to ensure optimal outcomes 182, 183. Despite challenges in overcoming safety, specificity, and broad-spectrum activity, personalized senescence-inducing therapies show great potential in the treatment of cancer and age-related diseases. Many of these senolytic drugs were initially developed as targeted anti-cancer agents, leveraging the overlap in signaling pathways between tumor and senescent cells 117. With continued research and clinical trials, personalized therapies for inducing cellular senescence are poised to become a significant direction in the future of cancer and age-related disease treatment.

### Long-term outcomes and safety considerations

Many questions remain to be addressed before launching large-scale human trials using either senolytics or senostatics or both. These include determining the optimal dosage, timing, and duration of treatment (i.e., intermittent vs. continuous) 184. Additionally, establishing endpoints for evaluation, such as monitoring biomarkers of senescence and the functional efficacy of the therapy, is critical 185.

Alvespimycin (17-DMAG), an HSP-90 inhibitor, has been shown to reduce normal tissue damage following radiation exposure without compromising the effectiveness of radiotherapy 53, 186. Similarly, the Bcl-2 family inhibitor navitoclax has demonstrated efficacy in improving radiation-induced pulmonary fibrosis 187, 188, mitigating radiation-induced hematotoxicity, and alleviating age-related hematopoietic stem cell dysfunction 121. Furthermore, navitoclax has been effective in delaying malignant glioma recurrence by eliminating radiation-induced senescent astrocytes 87. Navitoclax's potential to mitigate normal tissue radiation damage while enhancing radiation cytotoxicity in tumors is also supported by its ability to overcome hypoxia-driven radioresistance 189. Navitoclax (ABT-263) is an investigational BCL-2/BCL-xL inhibitor, and dose-dependent thrombocytopenia has been consistently reported as a principal dose-limiting toxicity in early oncology trials 53.

As with many current cancer therapeutics, the most promising approach to using senolytics may be combinatorial. While initially suppressing tumors, the chronic inflammation from the SASP poses significant risks 190. Persistent SASP factors can remodel the tumor microenvironment, promoting fibrosis, chronic inflammation, and secondary malignancies 191, 192. In breast cancer, for example, senescent cells alter the stroma, fostering a pro-tumorigenic environment 193. To mitigate these risks, therapies targeting senescent cells post-radiotherapy are being explored. Senolytic agents like Navitoclax selectively eliminate senescent cells, reducing SASP-related adverse effects 194. Combining immune checkpoint inhibitors with radiotherapy can enhance anti-tumor immunity and counteract the effects of SASP 148. Understanding the mechanisms, particularly the ATM and p53 pathways, is crucial for optimizing treatments and minimizing long-term risks 21, 82, 195.

### Therapeutic windows and trial-design considerations for RT-senescence targeting

A major translational challenge is defining a clinically actionable therapeutic window for senescence-targeted interventions after radiotherapy. Conceptually, the post-RT trajectory can be bifurcated into an acute phase (hours-days) dominated by DNA damage responses and immunogenic stress signaling, an early phase (days-weeks) when senescent-cell burden and SASP programs become measurable while immune surveillance remains operative, and a chronic phase (weeks-months and beyond) characterized by persistent senescent-cell accumulation, stromal/vascular niche remodeling, and late toxicities including fibrosis 2, 55, 56, 81. This staging underscores why senescence targeting must be schedule-dependent: premature, indiscriminate elimination risks blunting the initial pro-immunogenic effects of transient senescence, whereas delayed intervention may fail to preempt irreversible SASP-driven tissue remodeling 53, 54, 160.

Within this framework, senolytics may be most efficacious following the completion of a radiotherapy course—or after specific hypofractionated triggers—to debulk the senescent reservoirs that fuel late-onset inflammation 122, 127, 134. Conversely, senomorphics (SASP modulators) may be better positioned as a maintenance strategy to dampen chronic pro-fibrotic signaling while preserving the tumor-suppressive benefits of growth arrest 89, 122.

To translate these concepts into clinical practice, trial designs should transition from fixed calendar-based dosing toward biomarker-triggered, adaptive schedules 54. In this paradigm, treatment initiation and duration are guided by longitudinal liquid biopsy readouts, such as circulating SASP factors, extracellular vesicle-associated signatures, or senescence-specific epigenetic markers in cell-free DNA 25, 44, 66, 67. Such dynamic monitoring enables the identification of the physiological peak of senescence accumulation, allowing for precise 'on-target' intervention 58, 164.

Furthermore, early-phase trials must prioritize safety alongside radiotherapy-specific outcomes, incorporating rigorous monitoring for known toxicities including Bcl-xL-mediated thrombocytopenia 121, 127, 188. Special attention should also be directed toward the potential amplification of immune-related adverse events when these agents are integrated with immune checkpoint inhibitors, ensuring that the therapeutic window maximizes normal-tissue protection without compromising antitumor immunity 154, 179.

## Conclusion

Several unresolved questions will define the next phase of the field: (i) whether heterogeneity among senescent cells—determined by their origin, the context of damage, and their spatial niche—governs SASP composition and eventual fate, such as stable arrest, escape, or immune clearance; (ii) how the kinetics and efficiency of senescent-cell clearance in humans vary after radiotherapy across different tissues, fractionation schedules, and immune states; and (iii) how to design intelligent, conditional delivery systems, including activatable nanoparticles, antibody-drug conjugates, or prodrug strategies, that can selectively target senescent cells within tumors while sparing normal tissues.

Translationally, future clinical development must integrate patient stratification based on baseline frailty, age, comorbid inflammatory burden, organ-at-risk dose metrics, and early post-radiotherapy SASP/DDR biomarker kinetics to determine which patients to treat, the optimal intervention timing, and the most suitable senescence-targeted modality, whether senolytics, senomorphics, or immune clearance. The establishment of robust biomarkers, defined treatment windows, and comprehensive safety profiles will be crucial to successfully translate senescence-targeted combinations into durable tumor control while minimizing long-term toxicity.

## Figures and Tables

**Table 1:** Representative studies investigating radiotherapy-induced cellular senescence and tumor microenvironment modulation

Author (Year)	Model & regimen/context	Predominant senescent compartment	Key TME findings	Implication
Ji et al. (2024) 68	Glioblastoma (GBM); preclinical cranial RT	Senescent astrocytes (TME)	Astrocyte senescence; SASP; myeloid recruitment; immunosuppressive TME	Astrocyte senescence promotes post-RT regrowth; targeting senescent astrocytes may reduce recurrence
Fletcher-Sananikone et al. (2021) 87	GBM; fractionated cranial RT	Senescent astrocytes/stromal cells	Stromal senescence accumulation; supports residual tumor survival; relapse-permissive niche	Post-RT navitoclax (ABT-263) reduces recurrence; supports a defined post-RT senolytic window
Meng et al. (2021) 92	NSCLC; thoracic RT (tumor control + lung fibrosis)	Senescence-like fibroblasts/stroma	Profibrotic SASP; fibroblast activation; fibrosis; tumor-protective microenvironment	Stromal senescence links fibrosis and radioresistance; stromal senolytics may widen the therapeutic window
Su et al. (2021) 81	Radiation-induced pulmonary fibrosis model; thoracic irradiation	Senescent macrophages	Macrophage SASP; fibroblast activation; chronic inflammation maintenance	Senescent immune cells drive late normal-tissue toxicity; selective clearance may mitigate RT-induced fibrosis
Ruscetti et al. (2020) 74	Pancreatic cancer; therapy-induced senescence (TIS)	Senescent tumor + endothelial/stromal cells	SASP-driven vascular abnormality; ECM remodeling; invasion-permissive niche	Vascular/stromal senescence creates vulnerabilities yet promotes progression; rationale for co-targeting senescence and vasculature with RT
Ruhland et al. (2016) 95	Transplantable tumor models; stromal senescence	Senescent fibroblasts/stroma	Senescent stroma; myeloid recruitment; immunosuppressive, tumor-promoting niche	Stromal senescence alone can drive tumor growth and immune suppression; supports targeting stromal senescence as an RT-relevant strategy

**Abbreviations:** RT, radiotherapy; TME, tumor microenvironment; SASP, senescence-associated secretory phenotype; GBM, glioblastoma; NSCLC, non-small cell lung cancer; ECM, extracellular matrix.** Note:** Senescence markers and RT dosing schedules are reported in the original studies.

**Table 2:** Therapeutic strategies to modulate radiotherapy-induced cellular senescence and SASP

Strategy category	Representative examples	Core action (senescence / SASP)	RT-related rationale / implication
Senolytics (broad-spectrum)	Cardiac glycosides (Digoxin/ Ouabain) 117.	Induce apoptosis in senescent cells; SASP burden↓	**Timing:** Post-RT (days-weeks); clear accumulated tumor/normal-tissue senescent cells to reduce late inflammation/toxicity 53, 122.
Senolytics (Bcl-2 family-targeted)	Navitoclax (ABT-263) 121, 188, 189.	Bcl-2/Bcl-xL inhibition; preferential killing of therapy-induced senescent cells	**Timing:** Post-RT window; mitigates RT-induced fibrosis/haematotoxicity 188 and may delay GBM recurrence by eliminating senescent astrocytes 87. **Limitation:** thrombocytopenia (dose-limiting).
Senolytics (flavonoids / combinations)	Dasatinib + quercetin (D+Q) 119; fisetin 137.	Disrupt senescent-cell pro-survival pathways; senescent-cell burden↓	**Timing:** Post-RT (or intermittent dosing); practical oral regimens to attenuate chronic SASP-driven effects, particularly in older/frail patients 127.
Senostatics / SASP modulators (mTOR, metabolism)	Rapamycin; metformin 124, 126.	mTOR/metabolic modulation; SASP dampening / reprogramming (SASP↓)	**Timing:** Maintenance after RT; limit chronic inflammation/fibrosis in normal tissues while preserving initial tumor-suppressive senescence.
Senostatics / SASP modulators (JAK-STAT, NF-κB)	JAK inhibitors (e.g., ruxolitinib); NF-κB pathway inhibitors 56, 72.	Block key SASP transcriptional programs (IL-6/IL-8/chemokines); bystander senescence↓	**Timing:** Post-RT / maintenance; reduce SASP-driven immune suppression and late toxicity risk (lung/heart/brain) 53.
Immunotherapy targeting senescent cells	ICIs (anti-PD-1/PD-L1; anti-CTLA-4) 57, 154, 179; engineered immune effectors (CAR-T-like strategies) 134; NK-based strategies 180	Enhance immune recognition and clearance of senescent tumor/stromal cells	**Timing:** RT → immunotherapy sequencing; use RT as senescence inducer/immunogenic priming, then eliminate senescent cells to convert transient control into durable tumor control.
Agents mitigating RT-induced normal-tissue senescence	HSP90 inhibitor alvespimycin (17-DMAG) 144, 186; navitoclax 188	Reduce accumulation/survival of senescent cells in irradiated normal tissues	**Timing:** Organ-protection strategy (peri-RT or early post-RT); widen therapeutic window by reducing late fibrosis/organ dysfunction without compromising anti-tumor efficacy.
RT optimisation to modulate senescence burden	FLASH-RT 4, 5; proton/heavy-ion RT 45; optimised fractionation	Modify dose-rate/LET/fractionation to spare normal tissue; normal-tissue senescence/SASP↓	**Timing:** Upfront (planning stage); backbone approach enabling safer combinations with senolytics/senostatics.
Combined approaches (RT + senescence-targeted therapy)	RT + senolytics 53; RT + senostatics 122; RT + immunotherapy 188	Multi-layer control: senescent-cell burden↓; SASP↓; clearance↑	**Timing:** Sequential or adaptive regimens; maximise anti-tumor senescence while minimising pro-tumorigenic and toxic sequelae.

**Abbreviations:** RT, radiotherapy; SASP, senescence-associated secretory phenotype; GBM, glioblastoma; ICI, immune checkpoint inhibitor; LET, linear energy transfer.
